# Social Behavioral Deficits Coincide with the Onset of Seizure Susceptibility in Mice Lacking Serotonin Receptor 2c

**DOI:** 10.1371/journal.pone.0136494

**Published:** 2015-08-26

**Authors:** Julien Séjourné, Danielle Llaneza, Orsolya J. Kuti, Damon T. Page

**Affiliations:** 1 Department of Neuroscience, The Scripps Research Institute, 130 Scripps Way, Jupiter, Florida, 33458, United States of America; 2 Department of Brain and Cognitive Sciences and Picower Institute for Learning and Memory, Massachusetts Institute of Technology, 77 Massachusetts Avenue, Cambridge, Massachusetts, 02139, United States of America; Osaka University Graduate School of Medicine, JAPAN

## Abstract

The development of social behavior is strongly influenced by the serotonin system. Serotonin 2c receptor (5-HT_2c_R) is particularly interesting in this context considering that pharmacological modulation of 5-HT_2c_R activity alters social interaction in adult rodents. However, the role of 5-HT_2c_R in the development of social behavior is unexplored. Here we address this using *Htr2c* knockout mice, which lack 5-HT_2c_R. We found that these animals exhibit social behavior deficits as adults but not as juveniles. Moreover, we found that the age of onset of these deficits displays similar timing as the onset of susceptibility to spontaneous death and audiogenic-seizures, consistent with the hypothesis that imbalanced excitation and inhibition (E/I) may contribute to social behavioral deficits. Given that autism spectrum disorder (ASD) features social behavioral deficits and is often co-morbid with epilepsy, and given that 5-HT_2c_R physically interacts with Pten, we tested whether a second site mutation in the ASD risk gene *Pten* can modify these phenotypes. The age of spontaneous death is accelerated in mice double mutant for *Pten* and *Htr2c* relative to single mutants. We hypothesized that pharmacological antagonism of 5-HT_2c_R activity in adult animals, which does not cause seizures, might modify social behavioral deficits in *Pten* haploinsufficient mice. SB 242084, a 5-HT_2c_R selective antagonist, can reverse the social behavior deficits observed in *Pten* haploinsufficient mice. Together, these results elucidate a role of 5-HT_2c_R in the modulation of social behavior and seizure susceptibility in the context of normal development and *Pten* haploinsufficiency.

## Introduction

Serotonin (5-HT) is a key neurotransmitter that appeared early in evolution [1] and influences a variety of social processes [2] across species, from humans [3] to primates [4], rodents [5] and flies [6]. The serotonin receptor 5-Ht_2c_R, encoded by the *5Htr2c* gene, is a G protein-coupled receptor (GPCR) that is coupled to G_q_/G_11_ and modulates cellular excitability [7]. Pharmacological studies in animal models have demonstrated roles for 5-Ht_2c_R in communication [8, 9] and social interaction. For example, activation of 5-Ht_2c_R by mCPP [10, 11], a non-selective agonist, or by SSRI (selective serotonin reuptake inhibitor, indirect agonist via inhibition of the serotonin transporter SLC6A4) [12] reduces social interaction in rodents. Conversely, administration of the selective 5-Ht_2c_R antagonist SB 242084 in rats increases social interaction [11] and rescues social deficits caused by stress [13] or mCPP [10, 11].

Dysregulated activity of 5-HT_2c_R has been implicated in autism spectrum disorder (ASD), which features deficits in social interaction and communication (DSM-V, American Psychiatric Publishing, 2013). For example, hyperactivity of 5-HT_2c_R has been reported in two mouse models of ASD risk factors: in mice with chromosome 15q11-13 duplication due to overexpression of the 5-HT_2c_R-editing snoRNA MBII52 [14], and in mice with a loss of function mutation for methyl-CpG binding protein 1 [15]. In addition, there is evidence of a physical interaction between 5-HT_2c_R and Pten [16, 17], a negative regulator of the PI3-kinase pathway [18] and a risk factor for ASD [19–22]. However, it is not known how chronic alteration in 5-HT_2c_R activity during development affects social behavior. Thus, we examined the social behavior of juvenile and adult *Htr2c* knockout mice, which lack 5-HT_2c_R.

Interestingly, null mutant mice lacking 5-HT_2c_R are extremely susceptible to audiogenic seizures [23, 24], suggesting an underlying elevation of the ratio of cellular excitation to inhibition (E/I balance) in these mice. It has been speculated that social and cognitive deficits might arise from a modification in this E/I balance, for example, through increased activity in excitatory neurons or reduced inhibitory neuron activity [25–29]. Supporting this idea, elevation of the E/I balance in the prefrontal cortex in mice elicits a profound impairment of social behavior [30]. To examine this relationship, we also investigated the onset of audiogenic seizure susceptibility in *Htr2c* knockout mice.

## Material and Methods

### Animals

Strains used were B6.129-*Htr2c* ^*tm1Jke*^ [31] (from The Jackson Laboratory) and B6.129-*Htr2c* ^*tm1Jul*^ [24] (from The Jackson Laboratory). Both lines were crossed to a C57BL/6J background for at least 10 generations to reach congenicity. Mice of the *B6*.*129-Pten* ^*tm1Rps*^ line [32] were obtained from the repository at the National Cancer Institute at Frederick, where they were already backcrossed onto a congenic C57BL/6J background by the Donating Investigator. The line has been maintained by backcrossing to C57BL/6J mice for more than 10 generations. For behavioral experiments female *Htr2c* ^*tm1Jke/+*^ mice were crossed with C57BL/6J males, thus producing *Htr2c* ^*tm1Jke/Y*^ (referred to as *Htr2c* ^*-/Y*^) and *Htr2c* ^*+/Y*^ (referred to as wild-type) male offspring. For the analysis of spontaneous death, female *Htr2c* ^*tm1Jul/+*^ or *Htr2c* ^*tm1Jke/+*^ mice were crossed with *Pten* ^*tm1Rps/+*^ males, resulting in *Htr2* ^*+/Y*^, *Pten* ^*tm1Rps/+*^ (referred to as *Pten* ^*+/-*^) and *Htr2* ^*Jke/Y*^; *Pten* ^*tm1Rps/+*^ (referred to as *Htr2c* ^*-/Y*^; *Pten* ^***+/-***^) male offspring. No distinction is made between the *Htr2c* ^*tm1Jul*^ and *Htr2c* ^*tm1Jke*^ lines in the analysis of spontaneous death as they are phenotypically equivalent. Due to the localization of the *Htr2c* gene on the X chromosome and to random X chromosome inactivation, no female *Htr2c* ^*+/-*^ mice were used in this study to avoid complications arising from mosaicism.

Behavioral testing occurred between postnatal days 21 to 26 (P21–26) for juveniles and P85–90 for adults. All animals were housed in mixed-genotype groups of 2–5 mice per cage, with no differences in housing between genotypes. Food and water were provided *ad libitum* and animals were kept on a reversed 12-h light/dark cycle. All behavioral testing was performed during the dark (active) phase of the light cycle. Experiments were performed in accordance with National Institute of Health and Association for Assessment and Accreditation of Laboratory Animal Care guidelines and approved by The Scripps Research Institute’s Institutional Animal Care and Use Committee.

### Three-chamber social approach and social novelty

Juvenile and adult mice were tested as previously described [33, 34] under white-light conditions. Briefly, test mice were each placed into the center of a black acrylic center arena (60 x 30 x 30 cm) that was divided into three equal compartments (each 20 x 30 x 30 cm). Mice were habituated to the empty arena for 5 min on each of the two days prior to testing. The test day consisted of three phases: 5 min acclimation to the empty arena, 10 min sociability testing [choice between two acrylic tubes (20 cm tall, 10 cm in diameter, with 16 ¼” diameter holes in the bottom half of the cylinder), one containing a novel, same-sex conspecific (location counterbalanced across mice), the other being empty], and 10 min social novelty testing (novel, same-sex conspecific placed in the previously empty tube). Tubes and chambers were cleaned with quatricide and paper towel-dried between mice. Ethovision (Noldus Information Technology, Wageningen, The Netherlands) was used to score the time spent in each chamber, as well as the velocity and distance traveled, for each mouse. Different cohorts of mice were used for juvenile and adult experiments.

### Audiogenic seizure (AGS) testing

Juvenile (P25) and adult animals (P90) were moved to the testing area and left undisturbed for 1–3h prior to testing. AGS testing was performed using a Phenotyper box (29.2 x 29 x 30.5 cm, Pten-T10/N, Noldus Information Technology, Wageningen, The Netherlands) equipped with a speaker and clear walls, each inside a noise-attenuating box with fans on. Behavior was monitored via a CCD camera mounted on the ceiling of the box and recorded by Ethovision. After a 30 s period of acclimation to the chamber, a 108-dB white noise stimulus was maintained for 60 s or until overt seizure had occurred. Mice that exhibited no sign of seizure during the stimulus were then monitored for 30 min. The motor response to audiogenic stimulus was classified as described previously [35]: no response (NR), wild running (WR), clonic seizure (CS), tonic seizure (TS), respiratory arrest/death (RA).

### Lifespan Study

A total of 68 mice were used in the lifespan study with 20 wild-type mice, 21 *Pten* ^*+/-*^, *15 Htr2c* ^*-/Y*^; *Pten* ^*+/-*^ and 12 *Htr2c* ^*-/Y*^; *Pten* ^*+/-*^. Mice were maintained in standard conditions with 5 mice per cage and were permitted to live out their lives until death due to natural causes. The mice used in the lifespan study were not disturbed except to check on the mice twice each day and were euthanized if any sign of distress was observed following AAALAC recommendations.

### Drug treatment

20 minutes prior to testing, mice tested for social approach with 5-HT_2c_R antagonist were given an intraperitoneal injection of SB 242084 (Sigma-Aldrich) diluted to 0.3mg/ml in a 10% (2-hydroxypropyl)-β-cyclodextrin (Sigma-Aldrich) in sterile 25mM citric acid vehicle, or equivalent volume vehicle. For all injections, care was taken to handle animals gently to minimize stress.

### Data analysis

Independent-sample *t*-tests were used to assess the effects of genotype (wild-type, *Htr2c* ^*-/Y*^) on behavior, and paired-sample *t*-tests were used to analyze chamber preferences for the three-chamber social approach (% time in mouse chamber *vs*. % time in empty tube chamber) and novelty test (% time in novel mouse chamber *vs*. % time in familiar mouse chamber) for each genotype. Additionally, approach-avoidance scores [time in chamber with a social stimulus minus time in chamber with the empty tube; [34, 36]] were calculated, and genotypes were compared using independent-sample *t*-tests (wild-type, *Htr2c* ^*-/Y*^) or two-way analyses of variance (drug: vehicle, 0.3mg/kg SB 242084; genotype: wild-type, *Htr2c* ^*-/Y*^). Kaplan-Meier survival analysis was used with Log-Rank test followed by pairwise comparison (Holm-Sidak) to analyze the data. For the pharmacogenetic study, planned comparisons between genotypes for each drug (vehicle: wild-type *vs*. *Htr2c* ^*-/Y*^, SB 242084: wild-type *vs*. *Htr2c* ^*-/Y*^) and between drug treatment for each genotype (wild-type: vehicle *vs*. SB 242084, *Htr2c* ^*-/Y*^: vehicle *vs*. SB 242084), were performed using independent-sample *t*-tests. In all cases, normality was assessed using Levene’s test. All statistics were performed using PASW 18 (IBM Corporation, Armonk, NY, USA), with significance set at *p*<0.05. All graphs represent mean +/- SEM.

## Results

**Fig 1:**
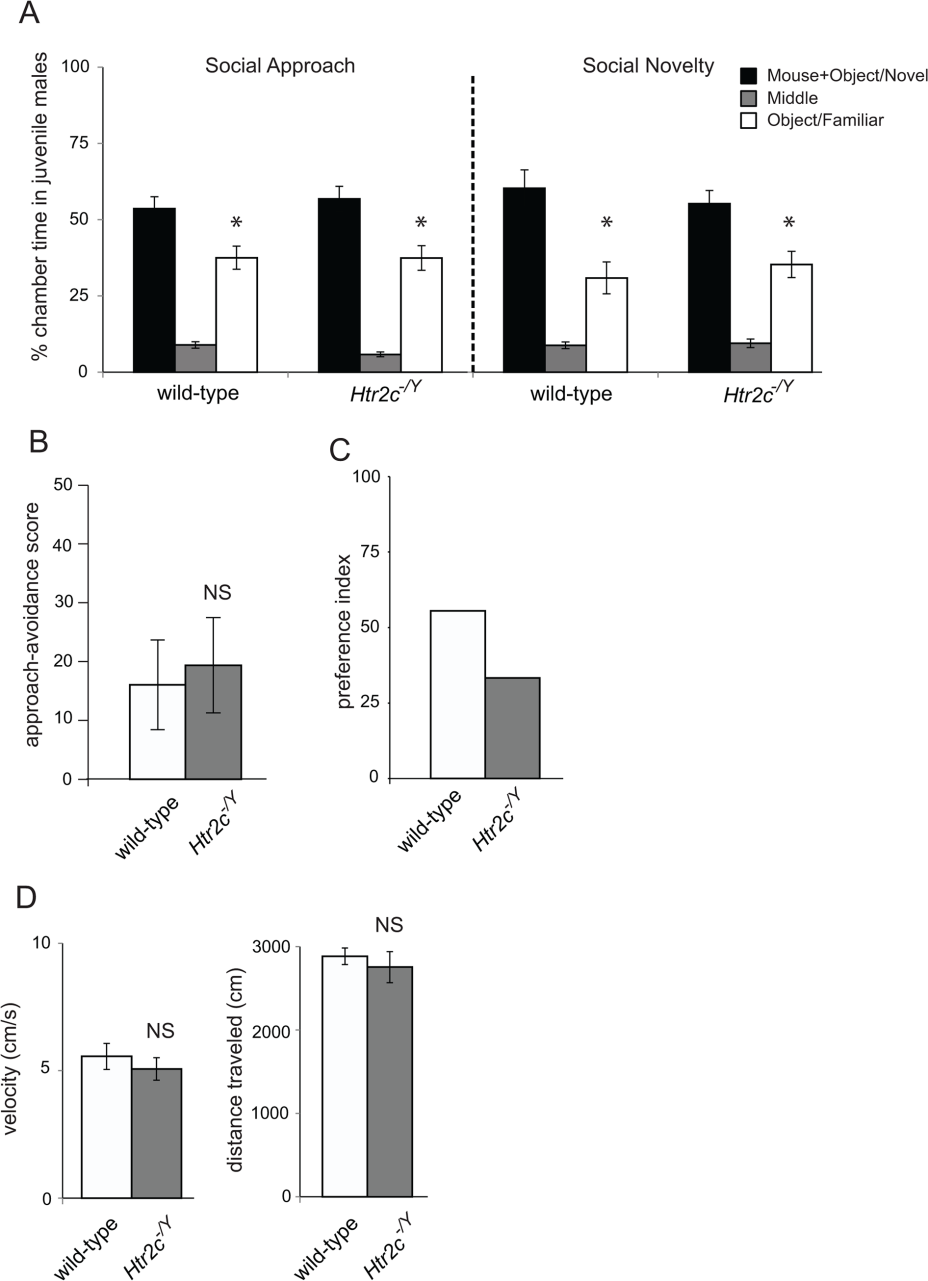
Three-chamber social approach and social novelty test in *Htr2c* ^*-/Y*^ juvenile male mice. **(A)** Time spent in each chamber. **(B)** Approach-avoidance scores. **(C)** Preference index: [(number of mice where the time in the mouse chamber was ≥ 10% than the time spent in the tube chamber)–(number of mice where the time in the mouse chamber was < 10% than the time spent in the tube chamber)]/(total number of mice). **(D)** Velocity and distance traveled. *n* = 9 per genotype. *: *p*<0.05, NS: non-significant difference with paired samples t-test **(A)** and independent-samples t-test **(B** and **D).**

We assessed the development of social behavior in *Htr2c* knockout mice using the three-chamber social approach and social novelty assay [33, 34] as juveniles (P21–26) or adults (P85–90). Both wild-type and *Htr2c* ^*-/Y*^ juvenile males spent significantly more time in the chamber with the novel social stimulus versus the chamber with the object control during the social approach assay (*t*(8) = 2.39, *p*<0.05 and *t*(8) = 2.35, *p*<0.05 respectively, Fig 1A left) and in the chamber with the novel social stimulus versus the chamber with the familiar social stimulus during the social novelty assay (*t*(8) = 2.34, *p*<0.05 and *t*(8) = 2.64, *p*<0.05 respectively, Fig 1A right). Correspondingly, we did not find a significant difference between wild-type and *Htr2c* ^*-/Y*^ juvenile mice for the approach-avoidance score (*t*(16) = 0.30, *p* = 0.77, Fig 1B). In order to determine the proportion of mice that show a strong preference for the mouse chamber, we designed a preference index, calculated as: (number of mice where the time in chamber 1 (containing stimulus mouse in cage) was ≥ 10% of the time spent in chamber 3 (containing empty cage))–(number of mice where the time in chamber 1 was < 10% of the time spent in chamber 3) / (total number of mice). Both wild-type mice (55.5%) and *Htr2c* ^*-/Y*^ mice (33.3%) presented a high positive preference index, showing that more than half of the mice had a strong preference for the mouse chamber (Fig 1C). Additionally, there was no significant difference in velocity or distance traveled in the social approach phase (*t*(16) = 0.73, *p* = 0.47 and *t*(16) = 0.62, *p* = 0.55 respectively, Fig 1D). These results suggest that *Htr2c* deletion does not impact social behavior of juvenile mice. Next, we wanted to test if constitutive deletion of *Htr2c* affects social behavior in adult mice.

**Fig 2:**
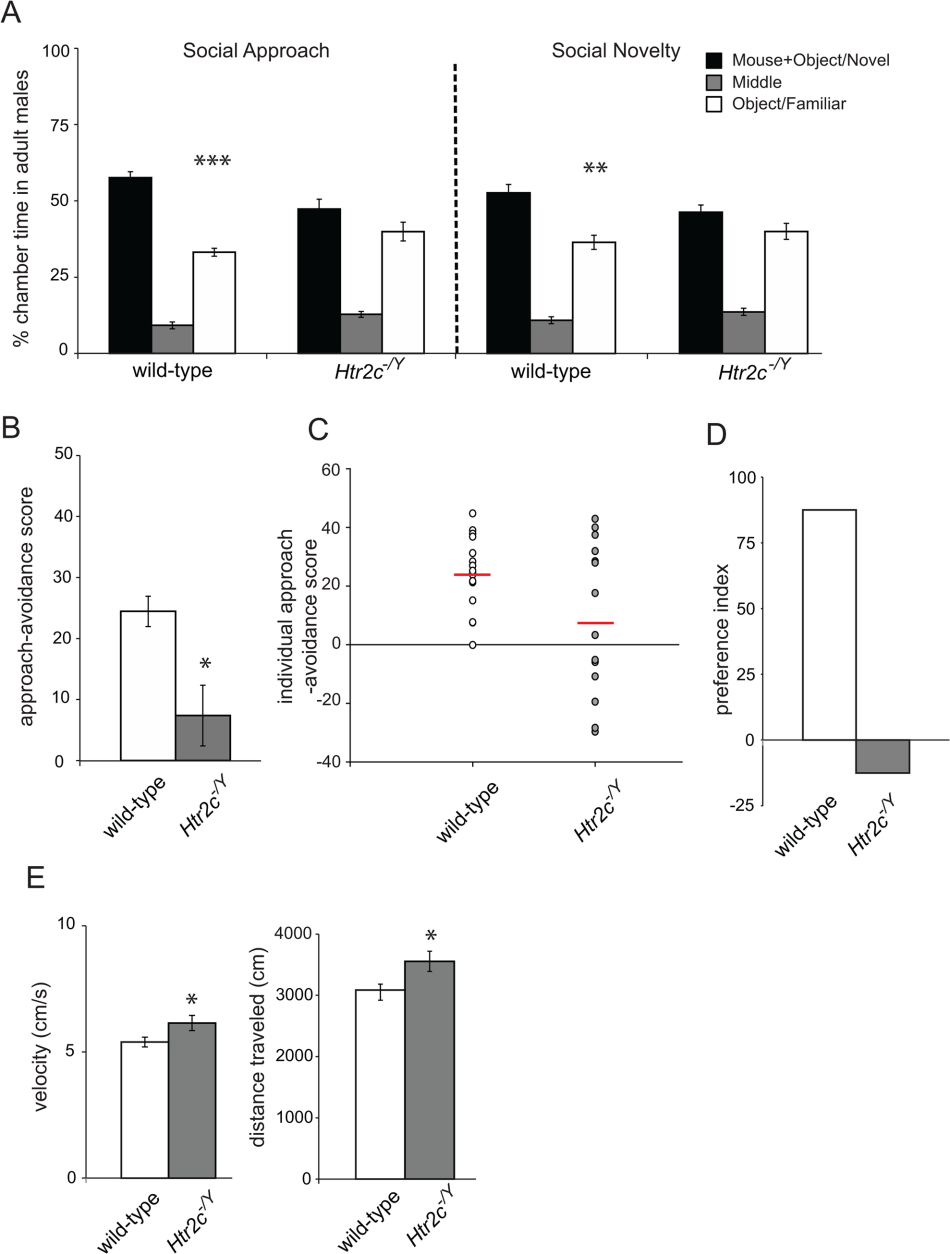
Three-chamber social approach and social novelty test in *Htr2c* ^*-/Y*^ adult male mice. **(A)** Time spent in each chamber. **(B)** Approach-avoidance scores. **(C)** Individual approach-avoidance scores of adult males. **(D)** Preference index: [(number of mice where the time in the mouse chamber was ≥ 10% than the time spent in the tube chamber)–(number of mice where the time in the mouse chamber was < 10% than the time spent in the tube chamber)]/(total number of mice). **(E)** Velocity and distance traveled. *n* = 16 per genotype. *: *p*<0.05, **: *p*<0.01, ***: *p*<0.001 with paired samples t-test **(A)** and independent-samples t-test **(B** and **E).**

Wild-type adult males spent significantly more time with the social stimulus during social approach (*t*(15) = 7.90, *p*<0.001, Fig 2A left), and with the novel social stimulus during social novelty (*t*(15) = 3.28, *p*<0.01, Fig 2A right), while *Htr2c* ^*-/Y*^ adult males showed no significant differences in chamber time during these assays (*t*(16) = 1.19, *p* = 0.25 and *t*(16) = 1.31, *p* = 0.21 respectively, Fig 2A). Analyzing these data using a social approach-avoidance score, we found that the time spent interacting with a stimulus mouse was significantly less in *Htr2c* ^*-/Y*^ than in wild-type mice (*t*(22.3) = 2.47, *p*<0.05, Fig 2B). The individual approach-avoidance score of *Htr2c* ^*-/Y*^ mice showed a bimodal distribution that was reflected by a higher variation in this score than in wild-type mice (Levene’s test for equality of variance: F = 14.91, *p*<0.01, Fig 2C). Using the preference index, we found a high preference index for wild-type mice (87.5%) while *Htr2c* ^*-/Y*^ mice showed a negative score (-12.5%; Fig 2D) confirming that less than half of these mice showed a preference for the mouse chamber. Moreover, we found that velocity and distance traveled in the social approach assay were significantly increased in *Htr2c* ^*-/Y*^ compared to wild-type mice (*t*(30) = 2.13, *p*<0.05 and *t*(30) = 2.46, *p*<0.05 respectively, Fig 2E) confirming previous studies [31, 37]. Thus, constitutive deletion of *Htr2c* decreased social interaction and increased locomotor activity in adult mice.

**Fig 3:**
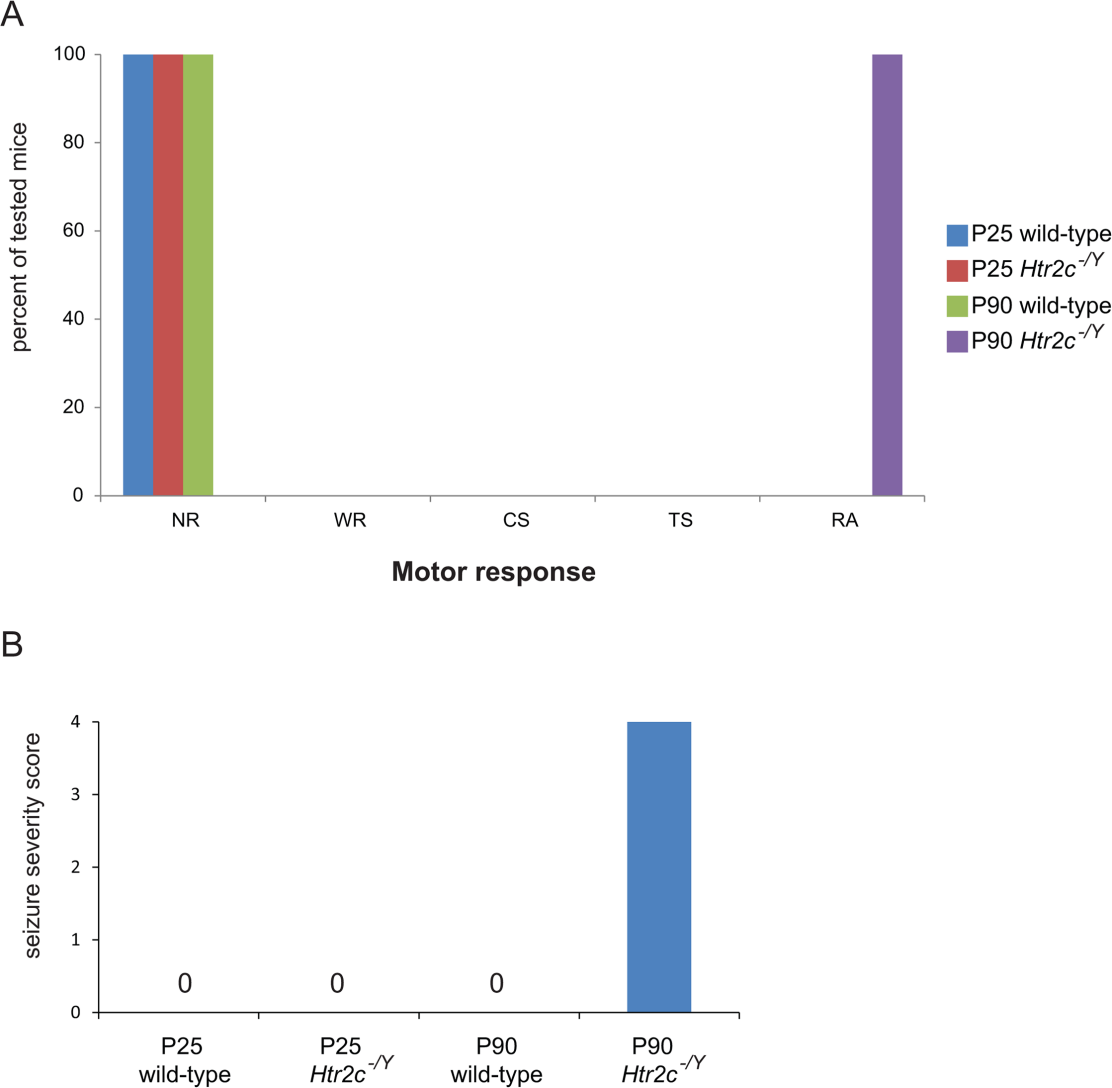
Audiogenic seizures in *Htr2c* ^*-/Y*^ adult male mice. **(A)** *Htr2c* ^*-/Y*^ adult mice (P90) show a severe response to audiogenic stimulus (RA: n = 16) while *Htr2c* ^*-/Y*^ juvenile mice (P25) or wild-type adult and juvenile did not exhibit any response (NR: n = 4, 16, 4 respectively). NR, no reponse; WR, wild running; CS, clonic seizure; TS, tonic seizure; RA, respiratory arrest/death. **(B)** The seizure severity score indicated a fully penetrant phenotype in adult *Htr2c* ^*-/Y*^ mice.

Adult *Htr2c* knockout mice feature spontaneous death and audiogenic seizures (AGS), which has been interpreted as reflecting a role for 5-HT_2c_R in tonic inhibition of excitability in neuronal networks [24, 38]. E/I imbalance within neural networks has been hypothesized as underlying social behavioral deficits in ASD and a variety of other neuropsychiatric disorders [25–28]. In light of this hypothesis, we find it interesting that adult, but not juvenile, *Htr2c* knockout mice display deficits in sociability. To further explore the relationship between social behavioral deficits and seizure susceptibility, we tested for AGS at P25 and P90, after social behavioral testing was complete in juvenile and adult cohorts of mice, respectively. No *Htr2c* ^*-/Y*^ mice displayed seizures at P25, confirming previous findings that juvenile *Htr2c* ^*-/Y*^ mice are not susceptible to AGSs [23]. At P90, we found that all *Htr2c* ^*-/Y*^ mice, but no wild-type mice, exhibited tonic-clonic seizures manifested by a brief forelimb and hindlimb flexion followed by a rigid and prolonged hindlimb extension (Fig 3). The latency to the tonic-clonic phase was extremely short, ranging from 2 to 6 seconds after the start of the white noise (average: 4.12 s). This confirms that the timing of social behavior deficits coincides with the onset of seizure susceptibility in *Htr2c* mice. It remains to be determined whether a causal relationship exists between these two phenotypes.

**Fig 4:**
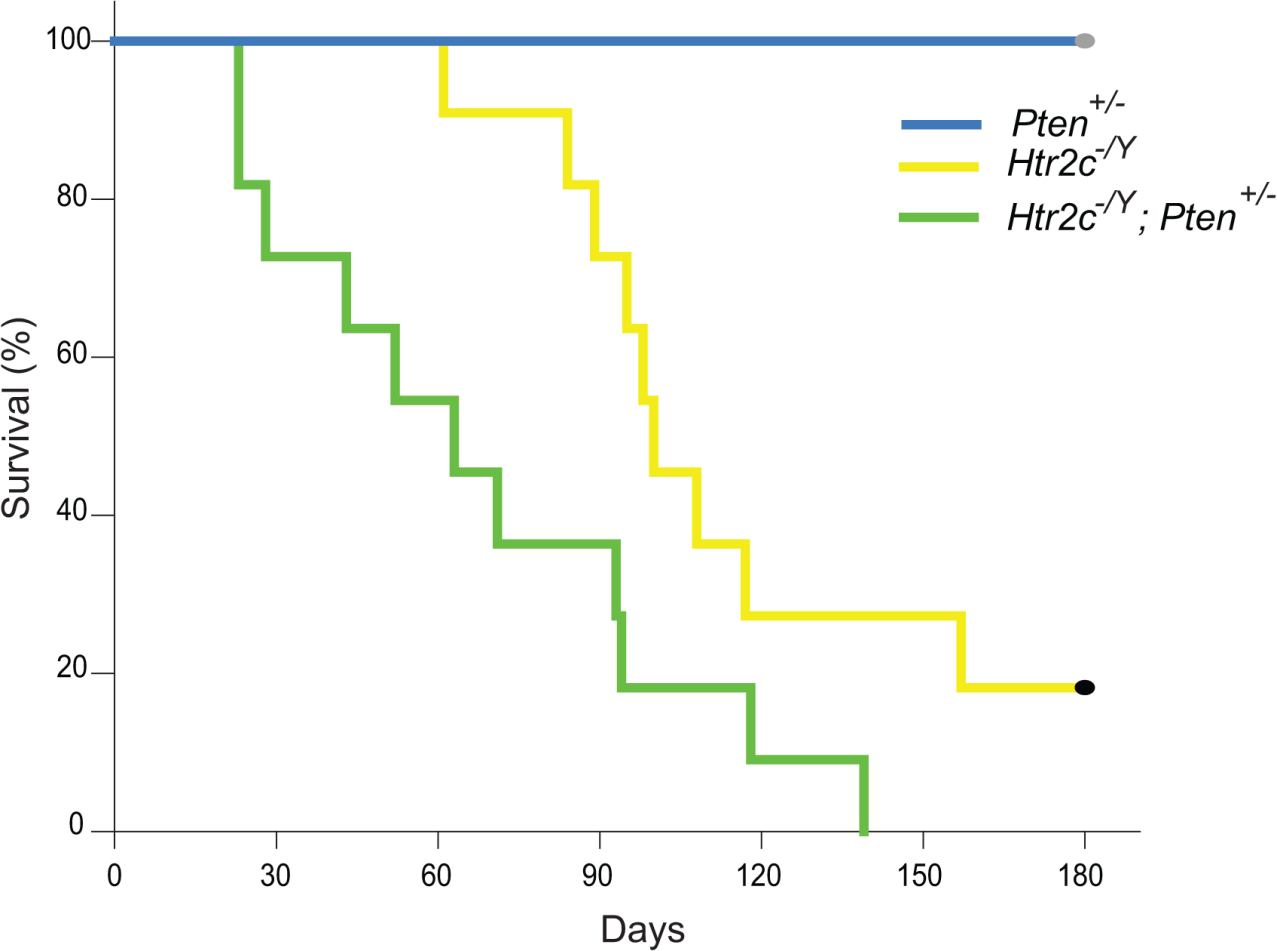
Spontaneous death in *Htr2c* ^*-/Y*^ mice is accelerated by a second-site mutation in *Pten*. Time course of spontaneous death in *Htr2c* ^*-/Y*^ (*n* = 15), *Pten* ^*+/-*^ (*n* = 21) and *Htr2c* ^*-/Y*^; P*ten* ^*+/-*^ (*n* = 12) mice.

*Pten* is a significant risk factor for ASD in humans [19–22] and various mouse models of *Pten* deletion show a deficit in social behavior [33, 34, 39, 40]. It has been shown previously that haploinsufficiency for the *Slc6a4* gene, encoding the serotonin transporter, can exacerbate the social behavior deficits observed in *Pten* ^*+/-*^ mice [34]. Moreover, Pten and 5-HT_2c_R receptor interact biochemically in dopaminergic neurons of the ventral tegmental area [17], making mutations in *Pten* a strong candidate for modifying the phenotypes observed in *Htr2c* ^*-/Y*^ mice. As *Htr2c* deletion did not affect the preference of the juvenile mice in the 3-chamber assay (Fig 1), we aimed to investigate the social behavior of *Htr2c* ^*-/Y*^ *; Pten* ^*+/-*^ adults. However, we found that only 20% of *Htr2c* ^*-/Y*^ *; Pten* ^*+/-*^ mice survived to P90 (Fig 4). This made adult social behavioral testing impractical in these animals. Wild-type (*n* = 20) and *Pten* ^*+/-*^ mice (*n* = 21) did not exhibit any spontaneous death within the timeframe examined (up to P180). An autopsy of mice that died spontaneously did not reveal any noticeable health problems such as hemorrhage, infarction, ischemia, hamartomas or tumors. Thus, as previously observed for *Htr2c* ^*-/Y*^ mice [24], we conjecture that the early lethality of *Htr2c* ^*-/Y*^ *; Pten* ^*+/-*^ mice may be attributed to an increase in spontaneous epileptic seizures. Using a Kaplan-Meier analysis, we found that there was a significant effect of genotype on survival (Log-Rank test: F(2) = 36, *p*<0.001). Pairwise comparisons revealed a significant difference on the survival of *Pten* ^*+/-*^ mice *vs*. *Htr2c* ^*-/Y*^ (*p*<0.001) and *Htr2c* ^*-/Y*^ *; Pten* ^*+/-*^ mice (*p*<0.001) and also between *Htr2c* ^*-/Y*^ and *Htr2c* ^*-/Y*^ *; Pten* ^*+/-*^ mice (*p*<0.05). We interpret these results as consistent with mutations in *Pten* and *Htr2c* interacting to influence spontaneous death, possibly via an exacerbation of the epileptic mechanism present in *Htr2c* knockout mice. However, because of the accelerated age of death, we were prevented from testing social behavior in these compound mutant animals.

**Fig 5:**
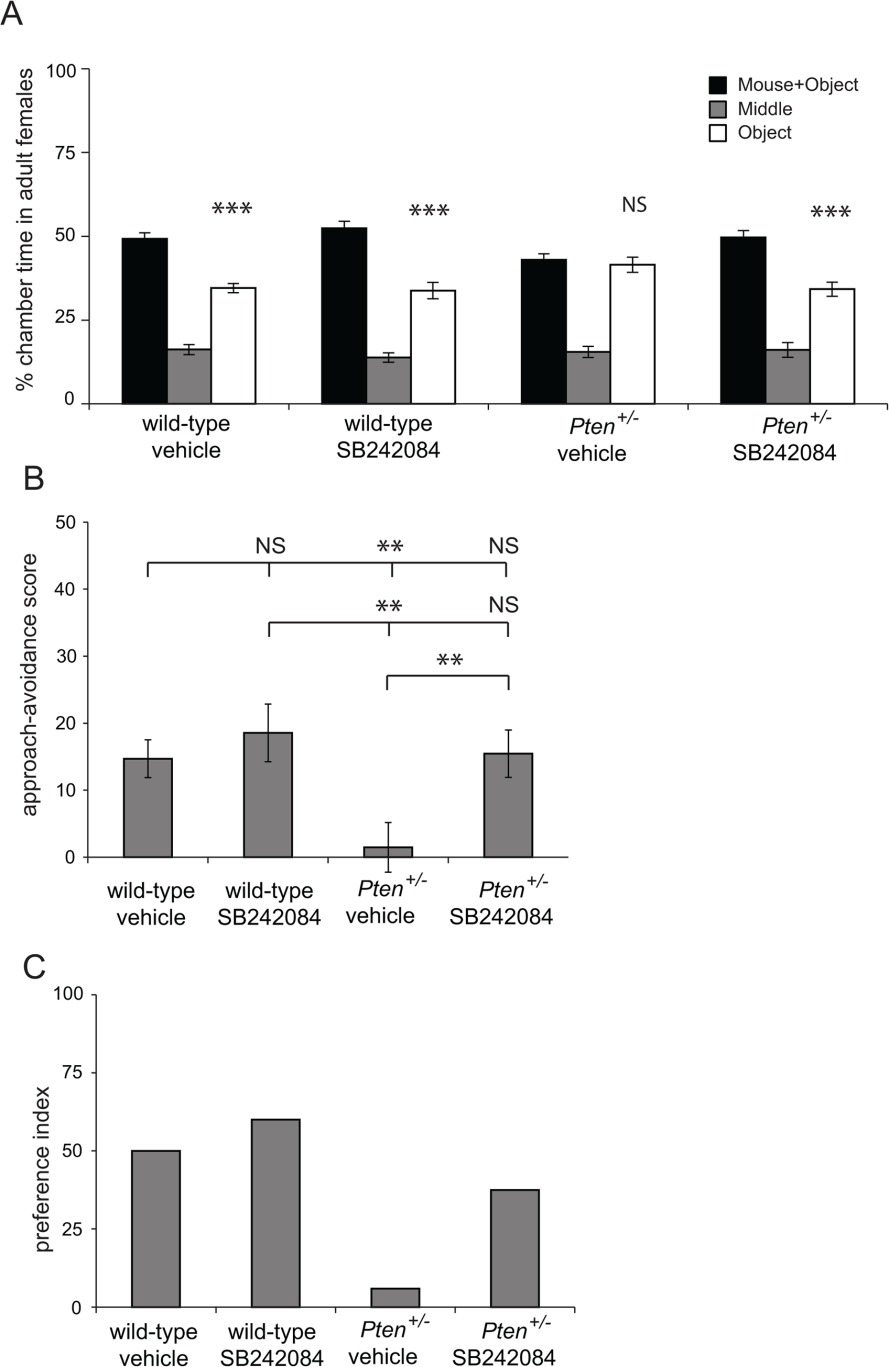
Rescue of social behavior deficits in P*ten* ^*+/-*^ mice by SB 242084 administration. Results of three-chamber social approach after administration of SB 242084 or vehicle to wild-type and *Pten* ^*+/-*^ adult female mice. **(A)** Time spent in each chamber. For vehicle treated groups, n = 16 wild-type and 17 *Pten* ^*+/-*^; for SB 242084-treated groups, n = 15 wild-type and 16 *Pten* ^*+/-*^. *** p < 0.001 using paired-samples t-test **(B)** Approach-avoidance score. ** p < 0.01 using planned comparisons. **(C)** Preference index.

As an alternative approach to explore whether Pten and 5-HT_2c_R interact to influence social behavior, we used SB 242084, a selective antagonist of 5-HT_2c_R that has been shown to increase social interaction behavior [11] and rescue social investigation deficits associated with stress [13] without increasing seizure susceptibility [11] in rodents. We used adult female *Pten* haploinsufficient and wild-type female mice as social approach deficits in female *Pten* ^*+/-*^ mice are well described [33, 34]. We administered 5-HT_2c_R antagonist SB 242084 systemically at 0.3 mg/kg, a dose that has been shown to significantly increase social interactions in rats [11, 13], and tested social approach behavior using a three-chamber social approach apparatus. Vehicle-treated wild-type animals spent significantly more time in the chamber containing the social stimulus mouse than the chamber containing an empty tube, while vehicle-treated *Pten* ^+/-^ mice did not display this preference (*t*(15) = 5.23, *p*<0.001 and *t*(16) = 0.40, *p* = 0.70, Fig 5A), indicating a deficit in sociability consistent with previous findings in untreated *Pten* ^+/-^ animals [33, 34]. Wild-type and *Pten* ^*+/-*^ mice treated with SB 242084 both displayed a significant preference for the chamber containing the social stimulus mouse (*t*(14) = 4.32, *p*<0.001 and *t*(15) = 4.38, *p*<0.001, Fig 5A). Analyzing these data using a social approach-avoidance score, we found a significant effect of genotype or SB 242084 treatment on the time spent to interacting with the stimulus mouse (Two-way ANOVA: Effect of genotype: F (1, 63) = 5.32, *p*<0.05. Effect of SB 242084 treatment: F (1, 63) = 6.32, *p*<0.05. Interaction between genotype and SB 242084 treatment: F (1, 63) = 1.97, *p* = 0.17 (n.s.), Fig 5B). Planned comparisons revealed a significant difference between *Pten* ^*+/-*^ mice treated with vehicle only and *Pten* ^*+/-*^ mice treated with SB 242084 (*t*(31) = 2.73, *p*<0.01) and between wild-type mice treated with vehicle and *Pten* ^*+/-*^ mice treated with vehicle (*t*(31) = 2.82, *p*<0.01), but no significant difference between wild-type treated with vehicle and wild-type treated with SB 242084 (*t*(29) = 0.76, *p* = 0.45) and between wild-type mice treated with SB 242084 and *Pten* ^*+/-*^ mice treated with SB 242084 (*t*(29) = 0.56, *p* = 0.58). Additionally, we found that, while *Pten* ^*+/-*^ mice treated with vehicle presented a very low preference index (5%), wild-type mice treated with vehicle or SB 242084 and *Pten* ^*+/-*^ mice treated with SB 242084 all presented a high positive preference index (50, 60 and 37.5% respectively). There was no spontaneous death or other evidence of seizures in *Pten* ^*+/-*^ mice treated with SB 242084. These results show that SB 242084 treatment suppresses the social behavior deficits exhibited by *Pten* ^*+/-*^ mice.

## Discussion

We report here that adult *Htr2c* null mutant mice present social behavior deficits and that these deficits are restricted to adult mice and are not present in juvenile mice. It has been hypothesized that dysregulated E/I balance may contribute to social behavioral deficits in neuropsychiatric disorders such as ASD [25–29] and consistent with this idea, it has been shown that elevation of cellular E/I balance within the mouse medial prefrontal cortex elicits an impairment of social behavior [30]. Evidence supports that E/I imbalance is also responsible for seizure susceptibility and spontaneous death in *Htr2c* mutant mice [24, 41, 42]. Interestingly, similar to social behavior deficits, *Htr2c* null mutant mice present a susceptibility to AGS only in adulthood [23, 24, 31] (and Fig 3). Given this, together with the observation that pharmacological antagonism of 5-HT_2c_R does not increase susceptibility to seizures and can increase social investigation in rodents [10, 11, 13], it is reasonable to speculate that E/I imbalance in adult *Htr2c* knockout mice is responsible for both social behavior deficits and susceptibility to spontaneous and audiogenic seizures. Similarly, E/I imbalance might be responsible for both social behavioral deficits and epilepsy in some ASD patients, as it has been shown that 30% of ASD patients can present a co-morbidity for epilepsy [43]. Alternatively, it is possible that mutations in *Htr2c* lead to an early dysregulation of growth and connectivity that results in abnormal neural circuitry going into the critical period for social learning, with social deficits not manifesting until after the critical period closes. It has been shown, for example, that social isolation that occurs in a critical period of 2 weeks after weaning alters prefrontal cortex function and myelination and these phenotypes are not reversible after reintroduction into a social environment [44]. For this hypothesis, dopaminergic neurons are a strong candidate cell type since they are important for social behavior [33, 45–49] and express 5-Ht_2c_R [50]. Future experiments using timed conditional deletions of *Htr2c* will help answer this question.

We also find that a second site mutation in *Pten* accelerates the spontaneous death rate observed in *Htr2c* ^*-/Y*^ mice [23, 24]. Consistent with previous reports in *Htr2c* ^*-/Y*^ mice [24], autopsy of these mice did not reveal any noticeable health problems suggesting that the early lethality of *Htr2c* ^*-/Y*^ *; Pten* ^*+/-*^ is caused by an increase in spontaneous epileptic seizures. An increased susceptibility to seizures has been reported in different models of *Pten* conditional deletion [39, 40, 51, 52], and ASD patients with a *PTEN* mutation can also present with epilepsy [53]. Although we did not observe any spontaneous seizures in germline *Pten* ^*+/-*^ mice, it is possible that *Htr2c* mutation uncovers a susceptibility masked by the C57BL/6 background, which is normally seizure resistant [54]. This enhancement of a seizure-prone phenotype in *Htr2c* ^*-/Y*^ *; Pten* ^*+/-*^ mice might seem surprising given that Pten physically interacts with 5-HT_2c_R to repress its activity [17], thus one might predict that *Pten* and *Htr2c* mutations would have opposing phenotypic effects. Indeed, we have demonstrated that pharmacological antagonism of 5-HT_2c_R with SB 242084 in *Pten* haploinsufficient mice is capable of restoring a preference for social investigation in a three-chamber social approach assay. In interpreting these results, it is worth considering that chronic treatment using a 5-HT_2c_R antagonist does not increase seizure susceptibility [11] and we did not observe any spontaneous seizures in *Pten* haploinsufficient mice treated with SB 242084, indicating that gross E/I balance is normal. Thus, it is possible that mutations in *Htr2c* and *Pten* may act in different cell types to influence E/I balance in a manner that is not recapitulated by pharmacological antagonism of 5-HT_2c_R. For example, mutations in *Htr2c* might result in decreased activity of GABAergic neurons [55–58] while *Pten* mutations result in increased activity of excitatory neurons [59], resulting in a synergistic elevation of E/I balance. Another possibility is that Pten and 5-HT_2c_R might have different periods and mechanisms of interaction: one complementary interaction in early development that sets up a later vulnerability to seizures, and another antagonistic interaction in mature circuitry that can reverse social behavioral deficits. Additionally, SB 242084 might rescue the social behavior deficits observed in *Pten* haploinsufficient mice through an anxiolytic effect, for example by modulating release of dopamine in the limbic system [60]. While the precise mechanism remains to be discovered, the above data are consistent with the hypothesis that 5-HT_2c_R interacts with Pten to modulate both E/I balance and the circuitry influencing social behavior. Important issues to address in future work will be to determine the degree to which modulating 5-HT_2c_R is significant for the ASD-therapeutic effects of drugs such as risperidone, fluoxetine and olanzapine, which target a broad range of molecules, including 5-HT_2c_R.
